# Estimating the Risk of Lower Extremity Complications in Adults Newly Diagnosed With Diabetic Polyneuropathy: Retrospective Cohort Study

**DOI:** 10.2196/60141

**Published:** 2025-05-29

**Authors:** Alyce S Adams, Catherine Lee, Gabriel Escobar, Elizabeth A Bayliss, Brian Callaghan, Michael Horberg, Julie A Schmittdiel, Connie Trinacty, Lisa K Gilliam, Eileen Kim, Nima S Hejazi, Lin Ma, Romain Neugebauer

**Affiliations:** 1 Departments of Health Policy and of Epidemiology and Population Health, School of Medicine, Stanford University, Encina Commons, 615 Crothers Way, Stanford, CA, 94305, United States, 1 6507247555; 2 Division of Research, Kaiser Permanente Northern California, Pleasanton, CA, United States; 3 Department of Family Medicine, University of Colorado School of Medicine, Institute for Health Research, Kaiser Permanente Colorado, Aurora, CO, United States; 4 Department of Neurology, School of Medicine, University of Michigan, Ann Arbor, MI, United States; 5 Mid-Atlantic Permanente Research Institute, Washington DC, MD, United States; 6 The Queen's Medical Center, Honolulu, HI, United States; 7 Kaiser Permanente South San Francisco, South San Francisco, CA, United States; 8 Kaiser Permanente Oakland Medical Center, Oakland, CA, United States; 9 Department of Biostatistics, Harvard T.H. Chan School of Public Health, Boston, MA, United States

**Keywords:** diabetes, neuropathy, machine learning, risk prediction, lower extremity complication, diabetic, AI, artificial intelligence, diabetic polyneuropathy, California, Colorado, USA, foot ulcer, logistic regression model, regression model, lower extremity

## Abstract

**Background:**

Diabetes-related lower extremity complications, such as foot ulceration and amputation, are on the rise, currently affecting nearly 131 million people worldwide. Methods for early detection of individuals at high risk remain elusive. While data-driven diabetic polyneuropathy algorithms exist, high-performing, clinically useful tools to assess risk are needed to improve clinical care.

**Objective:**

This study aimed to develop an electronic medical record–based machine learning algorithm that would predict lower extremity complications.

**Methods:**

We conducted a retrospective longitudinal cohort study to predict the risk of lower extremity complications within 24 months of an initial diagnosis of diabetic polyneuropathy. From an initial cohort of 468,162 individuals with at least 1 diagnosis of diabetic polyneuropathy at one of 2 multispecialty health care systems (based in northern California and Colorado) between April 2012 and December 2016, we created an analytic cohort of 48,209 adults with continuous enrollment, who were newly diagnosed with no evidence of end-of-life care. The outcome was any lower extremity complication, including foot ulceration, osteomyelitis, gangrene, or lower extremity amputation. We randomly split the data into training (38,569/48209; 80%) and testing (9,640/48209; 20%) datasets. In the training dataset, we used super Learner (SL), an ensemble learning method that employs cross-validation and combines multiple candidate risk predictors, into a single risk predictor. We evaluated the performance of the SL risk predictor in the testing dataset using the receiver operating characteristic curve and a calibration plot.

**Results:**

Of the 48,209 individuals in the cohort, 2327 developed a lower extremity complication during follow-up. The SL risk estimator exhibited good discrimination (AUC=0.845, 95% CI 0.826-0.863) and calibration. A modified version of our SL algorithm, simplified to facilitate real-world adoption, had only slightly reduced discrimination (AUC=0.817, 95%CI 0.797-0.837). The modified version slightly outperformed the naïve logistic regression model (AUC=0.804, 95% CI 0.783-0.825) in terms of precision gained relative to the frequency of alerts and number of patients that needed to be evaluated.

**Conclusions:**

We have built a machine learning–based risk estimator with the potential to improve clinical detection of diabetic patients at high risk for lower extremity complications at the time of an initial diabetic polyneuropathy diagnosis. The algorithm exhibited good discriminant validity and calibration using only data from the electronic medical record. Additional research will be needed to identify optimal contexts and strategies for maximizing algorithmic fairness in both interpretation and deployment.

## Introduction

Up to 50% of the more than 38 million Americans who have diabetes experience some peripheral nerve damage, known as diabetic polyneuropathy [1,2]. Distal symmetric polyneuropathy, the most common type of diabetic polyneuropathy, is frequently characterized as pain, tingling, and numbness that starts in the extremities. Motor and autonomic involvement, also seen in those with diabetic polyneuropathy, can lead to foot deformity. Lower extremity complications associated with diabetic polyneuropathy include foot ulceration, osteomyelitis, and gangrene, leading to amputation [1-4]. Diabetic polyneuropathy is the leading risk factor for the recent resurgence in nontraumatic lower extremity amputations in the United States [3,4], with health care costs falling between US $ 4.6 and US $ 13.7 billion dollars per year [1].

Although there is no cure for this condition, it may be possible to reduce the clinical impact of diabetic polyneuropathy through control of blood sugar levels, annual foot checks, patient education, and specialty referrals (eg, podiatry) [5]. Novel digital interventions may also hold promise for improving self-care and function among individuals at risk of lower extremity complications [6]. While likely not done often enough, identifying and referring high-risk patients for enhanced educational intervention may be cost-effective [7].

Both clinical and nonclinical factors may contribute to delayed diagnosis and undertreatment in subgroups of this patient population [8-10]. For example, differences in patient presentation or clinician interpretation of symptoms may be driven by clinical, interpersonal and societal factors. Additional tools are needed to reduce diagnostic uncertainty, as well as augment human factors to promote evidence-based care [8].

Several screening tools designed to facilitate early detection of diabetic polyneuropathy are currently used in clinical practice. These include monofilament tests, brief questionnaires (eg, the Michigan Neuropathy Screening Instrument) [11], and vibration testing, among others. Collectively, these instruments have been criticized for their lack of accuracy and vulnerability to human error and biases that lead to missed opportunities for follow-up [9,10].

Validated tools are needed to facilitate risk detection and reduce diagnostic uncertainty in the management of diabetic polyneuropathy [12-16]. However, the quality and transparency of existing risk stratification systems and algorithms is highly variable, limiting their use in everyday clinical practice [17]. The aim of this study was to evaluate the accuracy and clinical use of a machine learning (ML)–based algorithm designed to predict complications that develop within 2 years of an initial diabetic polyneuropathy diagnosis.

## Methods

### Creation of Analytic Cohort of Patients Newly Diagnosed With Diabetic Polyneuropathy

All data for this study were extracted from electronic medical records (EMRs) at 2 Kaiser Permanente regions, Northern California and Colorado, with facilities serving more than 5 million people. Patients with diabetes and related chronic conditions receiving care in these facilities are typically assigned to a single primary care provider with a robust panel management approach that leverages performance feedback, system-wide efficiencies, disease registries, and evidence-based practice [18].

We included clinicians engaged in diabetes quality improvement initiatives from 4 Kaiser Permanente regions and the University of Michigan Health System at each stage of the research endeavor to maximize the potential clinical use of the resulting algorithm [19].

The EMRs at both Kaiser Permanente health systems have automated patient files, which facilitate longitudinal observation of use and clinical assessments obtained across systems of care (eg, hospital, laboratory, pharmacy, and clinic). The Kaiser Permanente Northern California and Colorado embedded research units share a common data model for organizing EMR and administrative (claims) data for research use [20]. Data captured by the common data model include, but are not limited to, outpatient encounters, emergency department and inpatient claims, pharmacy orders and prescription fills, laboratory orders and results, and member enrollment and benefit coverage.

Using the EMRs, we identified 468,162 individuals who carried at least 1 diabetes-related diagnosis based on the *International Classification of Diseases, Ninth and Tenth Revisions *(*ICD-9* and *ICD-10*, respectively) between April 1, 2012, and December 31, 2016. From this group, we identified a subset of 121,619 individuals with confirmed diabetic polyneuropathy (ie, at least 1 inpatient or 2 outpatient *ICD-9* or *ICD-10* diagnoses within 12 months of each other) who were at least 18 years of age at the time of their first diagnosis. Measures of polyneuropathy severity were not available through the electronic health record. Therefore, clinicians on the study team reviewed the list of codes and discussed any disagreements on code inclusion before the list was finalized. Detailed codes used to identify diabetic polyneuropathy are included in Multimedia Appendix 1.

From the cohort of 121,619, we excluded 20,806 individuals who had 2 or more months of disenrollment from the health plan in the 24 months before the first diabetic polyneuropathy diagnosis (ie, start of follow-up) to reduce the likelihood of missing healthcare use data. Among the remaining 100,813, we excluded 51,728 individuals who had evidence of a possible previous diagnosis of diabetic polyneuropathy. Finally, we also excluded 876 individuals who received hospice or palliative care during the 24 months before diagnosis because these individuals may be less likely to receive usual standard care and would be unlikely to benefit from early identification of lower extremity complications. The resulting analytic cohort included 48,209 adults with newly diagnosed diabetic polyneuropathy.

### Identification of Lower Extremity Events and Predictors

We created a composite time-to-event outcome that included the 4 most common diabetic polyneuropathy-related lower extremity complications: foot ulceration (77.4%), osteomyelitis (6.53%), gangrene (5.46%), and nontraumatic lower extremity amputation (10.61%). We used evidence from earlier literature and clinician review to determine the diagnoses to be included [21-26]. A comprehensive list of codes used to identify lower extremity events is included in Multimedia Appendix 2 . We relied on the earlier peer-reviewed literature to define a standard phase-out period to account for the resolution of treatment for each type of event in order to distinguish between new and ongoing events [24].

### Identification of Candidate Predictors

A list of candidate covariates for predicting the outcome is presented in Multimedia Appendix 3. Drawing on the existing literature, we included several baseline characteristics (up to 24 months before the start of follow-up, ie, first diabetic polyneuropathy diagnosis) that were identified in previous studies as covariates associated with adverse diabetic polyneuropathy-associated events. These included self-reported demographics (age, sex, race, and ethnicity), clinical risk factors (HbA_1c_ levels, lipid levels, body mass index, comorbidity, blood pressure, use of specific medications [eg, insulin], smoking status, and alcohol use), specific comorbidities, (cardiovascular disease, peripheral artery disease, atrial fibrillation, heart disease, chronic pain, rheumatoid arthritis, sleep apnea, nondiabetic neuropathies, and previous falls), and other indicators of diabetes severity (number of different diabetes diagnoses, chronic kidney disease, cellulitis, diabetic retinopathy, stroke, Charcot foot, and previous diabetic polyneuropathy events) [12,13,17,26-28]. Several of these predictors, including laboratory results (blood pressure and cholesterol), use of health services (eg, durable medical equipment and diabetes medications), a Comorbidity Point Score [29] (based on the Centers for Medicaid and Medicare Services Hierarchical Condition Categories [score range: 0‐1014; scores >300 are rare]) and behavioral risk factors (eg, smoking and alcohol use) were not included in previous prediction algorithms used to estimate the risk of neuropathy-related outcomes [12].

For each covariate with missing values, we included a missing indicator variable and imputed missing values for continuous variables with the median value. Covariates with missing values are indicated as imputed in Multimedia Appendix 3. We note that baseline covariate values marked as unavailable are not included as “missing data” because we aim to predict outcomes using the type of data routinely available in health care databases, which are, by their nature, highly variable across patients and systems. Thus, we did not use advanced analytic methods to address bias concerns associated with missing data, as is typically warranted in causal inference problems (eg, multiple imputation).

### Algorithm Development and Validation

We developed an algorithm to predict lower extremity complications during the 24 months following an initial diagnosis of diabetic polyneuropathy. The first observed date of a diabetic polyneuropathy diagnosis served as the index date for the algorithm; this timepoint was defined as the start of patient follow-up and the clinical decision point when the constructed algorithm would be applied in practice. The first observed lower extremity complication was identified as the event date. We administratively censored follow-up after the eighth quarter (91-day interval) of follow-up (ie, at approximately 2 years).

To develop the algorithm, we combined data from the 2 health systems into a single dataset, then randomly split the data into distinct training (38,569/48209; 80%) and testing (9640/48209; 20%) datasets [30]. Selection of a risk predictor using the super learner (SL) ensemble learning methodology [31] was based solely on the results obtained using the training dataset; the independent validation set was used to evaluate the resulting discrimination and calibration performance. SL is a general loss-based ensemble learning method that uses cross-validation to combine [32] multiple candidate risk predictors defined by ML algorithms (eg, random forest) or parametric (eg, logistic) models into a single risk predictor referred to as “super learner”. The approach is grounded in statistical theory and its practical performance was demonstrated in previous applications [33].

Following the general approach of Polley and van der Laan [34], we used SL to construct a single point estimator of the vector of the discrete-time conditional hazards. The detailed mathematical formulas relating to the approach can been found in Multimedia Appendix 4. Implementation was generated using the sl3 R package (R Foundation for Statistical Computing) [35] with 10-fold cross-validation and the L2 loss function.

Due to potential limited computing infrastructures available to generate real-time predictions based on ML algorithms (eg, random forest) in some clinical settings, we also implemented a simplified version of the prediction approach described above in which we restricted the library of candidate predictors to a main-term logistic regression using training data pooled across all 8 quarters. This is equivalent to a classical discrete-time survival model with a logit link function [36]. The resulting predictor is thus a simpler function of predicted values from a single logistic regression that can be easily initiated in real-world clinical settings. In addition to this hazard-based logistic regression estimator for the cumulative incidence at 24 months, we also implemented a naïve estimator of the same cumulative incidence using a simple complete-case logistic regression in which outcomes from patients that were right-censored due to death or disenrollment from the health plan occurring before 24 months were treated as missing. We compared the performance [37] of the 3 estimators of the previously-described cumulative risks at 24 months (hazard-based SL, hazard-based logistic regression, and naïve logistic regression) using the areas under the receiver operating characteristic curves (AUCs) to evaluate sensitivity and specificity, calibration plots (ie, plots of observed versus predicted risks), and standard measures of predictive accuracy for a diagnostic test including sensitivity, specificity, positive and negative predictive value, and number needed to evaluate outcomes over a range of risk thresholds [38].

Finally, to determine whether the SL predictions were consistent with previous predictive approaches, we also examined select patient characteristics identified as predictive of risk in earlier studies by quintiles of the SL risk estimates for the 9640 patients in the testing data set. Based on the results from previous studies, the following characteristics were examined: age, HbA_1c_ levels, race and ethnicity, sex, evidence of select comorbid conditions known to be associated with lower extremity risk (ie, chronic kidney disease, heart failure, and peripheral artery disease), and a history of diabetic polyneuropathy events. We also examined differences in rates of symptoms by risk score quintile in the subset of patients in the test dataset who had been screened for diabetic polyneuropathy during the 12 months before their diagnosis using a single-item questionnaire (3644/9590; 38%). Data were extracted and formatted using SAS (version 9.4; SAS Institute) and analyses were performed in R (version 3.4.4).

### Ethical Considerations

This study was approved by the institutional review board at Kaiser Permanente Northern California. Kaiser Permanente Colorado ceded authority to the Kaiser Permanente Northern California Institutional Review Board. For this study, the requirement that informed consent and Health Insurance Portability and Accountability Act Privacy Rule authorization be obtained from study participants was waived. To protect the data and privacy of the patients included in our study, we limited the number of individuals who would have access to identifiable data. We removed personal identifiers from data that were transmitted outside each health system via a secure transfer site. Password-protected data were stored on the servers behind the firewalls maintained by each health system. Primary data were not distributed outside the 2 health care systems and only summary tables and figures were shared with external collaborators. In addition, we used randomly generated identifiers on all study documents, secured storage of digital data (computer files) on password-protected computers, and limited access to data with potentially identifying features to members of the research team working under the direction of the investigators for the duration of the project.

## Results

### Baseline Characteristics of Patients Meeting the Inclusion Criteria

**Figure 1.**
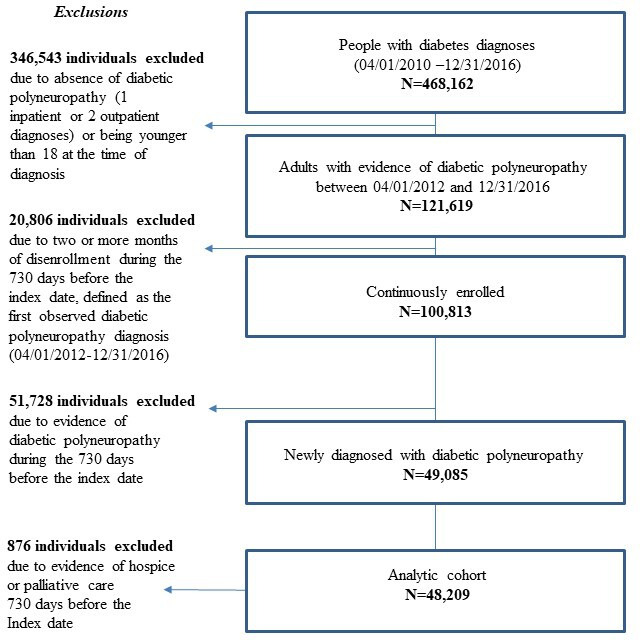
Cohort identification and selection criteria.

The cohort identification strategy is described in Figure 1; 48,209 adults newly diagnosed with diabetic polyneuropathy met the inclusion criteria for this study. At the time of their initial diabetic polyneuropathy diagnosis, characteristics of individuals in the training and testing datasets exhibited similar demographic and clinical characteristics. A comprehensive list of characteristics is presented in the Multimedia Appendix 3. Overall, the average age at the time of diabetic polyneuropathy diagnosis was 64 years (SD 12 years). In this cohort, 54% (25,828/48,209) were male and 52% (25,110/48,209) were of White race, 13% (6095/48,209) were of Asian race, 11% (5221/48,209) were of Black or African American race, 20% (9708/48,209) were of Hispanic or Latinx ethnicity, fewer than 2%(797/48,209) were of Native Hawaiian, Pacific Islander or Native American race and fewer than 3% (1278/48,209) had unknown race or ethnicity. The comorbidity point score [29,39], which was calculated using diagnosis records within 12 months previous the diabetic polyneuropathy diagnosis, was 36.91 (SD 31.05).

Among the 48,209 patients newly diagnosed with diabetic polyneuropathy in this study sample, 2327 (4.83%) developed a lower extremity complication during the 24-month follow-up period. A comparison of characteristics of those who did and those who did not develop a lower extremity complication during follow-up is presented in Multimedia Appendix 5.

### Discrimination and Calibration

**Figure 2.**
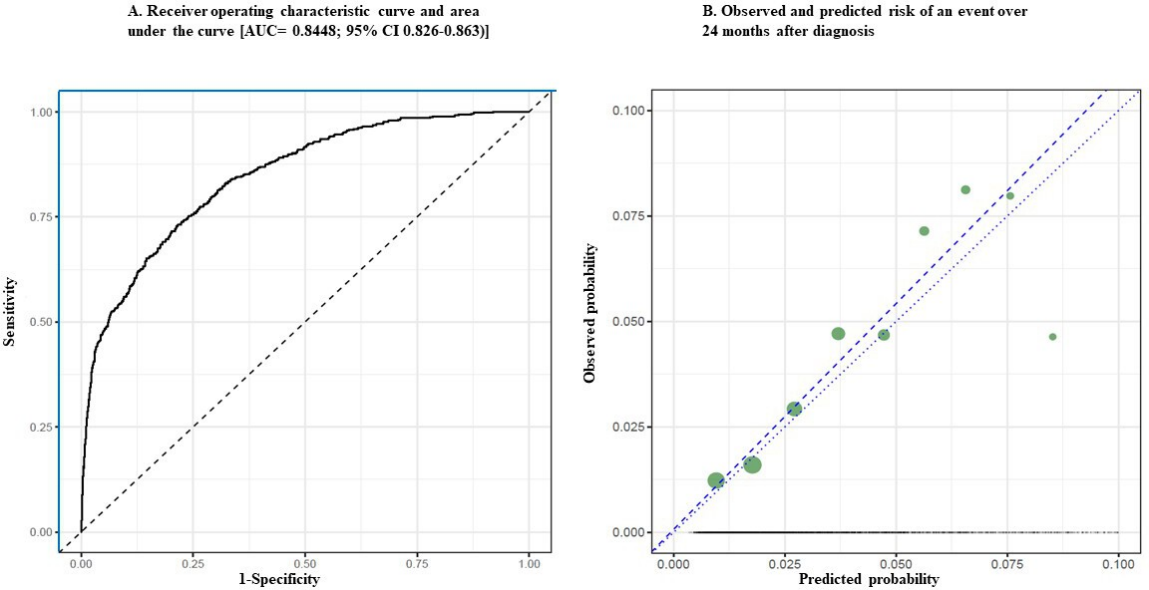
Discrimination and calibration plots for the model predicting hazard of lower extremity events among adults newly diagnosed with diabetic peripheral neuropathy (n=48,209). (A) Comparison of the model’s ability to distinguish between those at high and low risk compared with a perfect test and chance. The solid blue lines at the left and top borders represent a hypothetical test that perfectly distinguishes between high- and low-risk patients. The solid black curve displays the results of the predictive model, which correctly classifies patients with an event more often than it incorrectly misclassifies patients. The dashed line bisecting the graph represents a test that correctly classifies patients 50% of the time (ie, by chance). (B) Display of the observed and predicted probability of an event over the 24 months following an initial diagnosis of diabetic polyneuropathy. The dotted line represents a hypothetical model that perfectly predicts the event. The dashed line is the model-predicted risk at 24 months. The filled circles represent the number of actual events occurring over time.

As shown in Figure 2, the AUC statistic for the more complex SL was 0.845 (95% CI 0.826-0.863), indicating very good discrimination between high- and low-risk patients. Figure 2A compares the ability of the model to distinguish between those at high and low risk compared with a perfect test and chance. The solid blue lines at the left and top borders represent a hypothetical test that perfectly distinguishes between high and low risk patients. The solid black curve displays the results of the predictive model, which correctly classifies patients with an event more often than it incorrectly misclassifies patients. The dashed line bisecting the graph represents a test that correctly classifies patients 50% of the time (i.e., by chance). Figure 2B displays the observed and predicted probability of an event over the 24 months following an initial DPN diagnosis. The dotted line represents a hypothetical model that perfectly predicts the event. The dashed line is the model-predicted risk at 24 months. The filled circles represent the number of actual events occurring over time.

**Figure 3.**
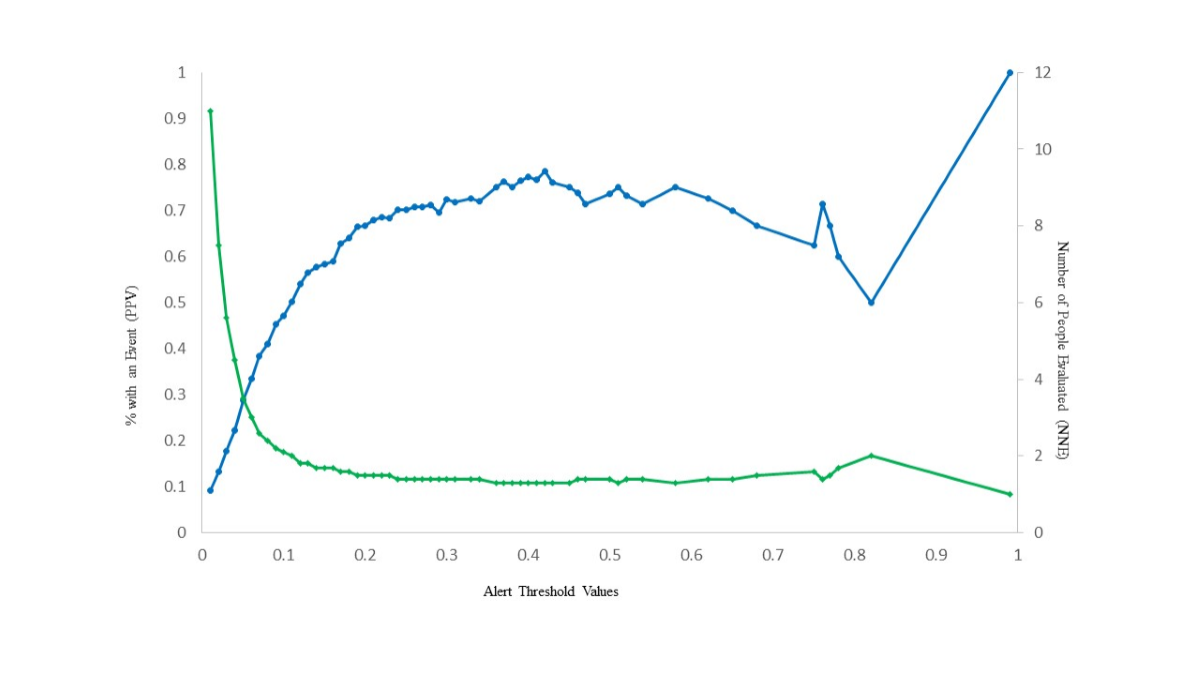
Positive predictive value and number needed to evaluate as a function of the decision threshold. The blue line with enclosed circles represents the percent with an event or the positive predictive value. The green line with enclosed diamonds represents the number needed to evaluate.

The SL predictor correctly classified patients who experienced an event as being at higher risk more often than it misclassified patients who did not experience an event as being at higher risk. Also shown in Figure 3 is a comparison of the observed with the predicted probability of a lower extremity complication over the 24 months following an initial diabetic polyneuropathy diagnosis. The blue line with circles represents the percent of people with an event or the positive predictive value. The green line with diamonds represents the number needed to treat. The algorithm demonstrated a high level of accuracy in predicting risk relative to perfect prediction.

The receiver operation characteristics curve for a simplified SL approach that included only one learner (logistic regression) was performed similarly to the more complex SL approach; the AUC for the simplified SL approach was 0.817 (95% CI 0.797-0.837). The similar performance indicated that a simplified logistic regression approach may be a viable alternative approach for use in clinical practice. By contrast, a naïve logistic regression model underperformed relative to both the complex and simplified SL approaches, with an AUC of 0.804 (95% CI 0.783-0.825).

To evaluate the use of these estimators for clinical decision-making, we compared the sensitivity, specificity, and the number needed to evaluate for varying risk thresholds at which an alert would be issued for the simplified SL compared with the naïve regression approach, as shown in Multimedia Appendix 6. Relative to a naïve logistic regression approach, the simplified SL approach yielded greater precision, triggered fewer alerts, and required fewer patients to be evaluated.

Figure 3 shows the positive predictive value relative to the number of patients needed to be evaluated as a function of the decision threshold. Setting an alert to trigger when an individual’s estimated risk reaches 30%‐50% using the simplified SL approach would yield a positive predictive value between 70%‐80%. Furthermore, we estimate that fewer than 2 patients would need to be evaluated to identify one likely to develop a lower extremity complication.

## Discussion

### Principal Findings

In this study, we leveraged rich clinical data from a longitudinal electronic health record to estimate the risk of developing lower extremity complications (ie, foot ulceration, osteomyelitis, gangrene, or amputation) among adults newly diagnosed with diabetic polyneuropathy. The resulting ML-enabled algorithm predicted risk of lower extremity complications with a high level of discriminant validity (AUC=0.845, 95%CI 0.826-0.863) and calibration. We concluded that a clinician would have to evaluate fewer than 2 patients newly diagnosed with diabetic polyneuropathy to identify one who would have a lower extremity event within the next 2 years.

### Comparison With Previous Work

Our algorithm performed better than a similar algorithm developed by Goyal et al [40] to identify infections in diabetic foot ulcers (AUC of 0.658). This difference may be due to our use of data from systems with a common data model, which reduces coding variability. Two other approaches reported accuracies for predicting diabetic foot and the severity of neuropathy that exceeded 0.9 [41,42] However, these studies used additional data, including clinical assessments and patient questionnaires that are not routinely documented in clinical practice.

Consistent with the earlier literature [12,13,40-43], our predictor identified several clinical subgroups that may be at higher risk for lower extremity complications, including individuals with poorer glycemic control, cardiovascular comorbidity, and previous lower extremity events. However, additional work will be needed to estimate both intended and unintended effects [44] of implementing the prediction algorithm in diverse care settings.

### Strengths and Weaknesses

Compared with previously published studies, strengths of our prediction approach include the use of a shorter time horizon (ie, 2-year time horizon), internal validation using an independent testing dataset, the inclusion of risk factors that are commonly available in EMRs, the use of a common data model [45], and the focus on overall risk of all lower extremity events rather than a single outcome (eg, foot ulceration).

Nonetheless, our prediction approach featured several important weaknesses. First, the predictor may be vulnerable to bias due to variability in the frequency of clinical monitoring during follow-up (ie, informative interval censoring). Second, our earlier studies identified differences in rates of diagnosis based on demographic factors (eg, age, race, and socioeconomic factors) [10]. This finding suggests that the predictor may underestimate risk in underserved populations. Additional research will be needed to evaluate the potential harm associated with the use of risk scores as well as other potential biases resulting from interval censoring [44].

### Future Directions

The diabetic polyneuropathy predictor developed using ML methods and electronic health data from a common data model demonstrated good calibration and a high level of predictive accuracy. Such a tool could be useful for identifying patients who might benefit from promising interventions. However, the use of this and similar prediction tools is limited by the availability of evidence-based practices and protocols to guide clinical decision making in response to risk information. Given the potential benefit of risk stratification of newly diagnosed patients, our results support the value of further research into how this might be implemented in clinical practice, including the potential unintended consequences of applying risk predictors in clinical practice and ensuring that these tools are applied equitably to the benefit of all patients.

### Conclusions

Diabetic polyneuropathy is a complex condition. There is not always a clear way to perform risk stratification for lower extremity complications associated with this disorder based on easily identifiable characteristics [2,5]. We hypothesize that diabetic polyneuropathy predictive analytics may be especially useful for identifying patients with diabetic polyneuropathy who are at highest risk for lower extremity complications soon after an initial diagnosis [8,17]. Nonetheless, technology-based prediction tools are not a panacea for complex clinical management. Consistent, strong evidence from diverse datasets and health care systems will be needed to determine the use of these and other strategies for patients, providers, and health systems in the context of real-world clinical practice [46].

## Supplementary material

10.2196/60141 Multimedia Appendix 1 List of *International Classification of Diseases, Ninth and Tenth Revisions* codes used to identify individuals with diabetic polyneuropathy.

10.2196/60141 Multimedia Appendix 2 Procedures used to identify the onset of lower extremity complications among adults newly diagnosed with diabetic polyneuropathy.

10.2196/60141 Multimedia Appendix 3 Characteristics of the study cohort.

10.2196/60141 Multimedia Appendix 4 Detailed description of algorithm development.

10.2196/60141 Multimedia Appendix 5 Comparison of predictor characteristics by event type.

10.2196/60141 Multimedia Appendix 6 Statistical performance of different risk prediction approaches.

## Abbreviations

AUC: area under the receiver operating characteristic curve
EMR: electronic medical record
*ICD-10*: *International Classification of Diseases, Tenth Revision*
*ICD-9*: *International Classification of Diseases, Ninth Revision*
ML: machine learning
SL: super learner
