# Supplementary material for: Estimating the Risk of Lower Extremity Complications in Adults Newly Diagnosed With Diabetic Polyneuropathy: Retrospective Cohort Study
Source: JMIR Diabetes. 2025 May 29;10:e60141. doi: 10.2196/60141 (PMC12140504; doi:10.2196/60141)
Supplement: Multimedia Appendix 1 [file diabetes-v10-e60141-s001.docx]

Appendix 1. List of ICD 9 and 10 codes used to identify individuals with diabetic polyneuropathy.

| **Version** | **Dx** | **Definition** |
| --- | --- | --- |
| 09 | 250.60 | Diabetes with neurological manifestations, type II or unspecified type, not stated as uncontrolled |
| 09 | 250.61 | Diabetes with neurological manifestations, type I [juvenile type], not stated as uncontrolled |
| 09 | 250.62 | Diabetes with neurological manifestations, type II or unspecified type, uncontrolled |
| 09 | 250.63 | Diabetes with neurological manifestations, type I [juvenile type], uncontrolled |
| 09 | 356.4 | Idiopathic progressive polyneuropathy |
| 09 | 356.8 | Other specified idiopathic peripheral neuropathy |
| 09 | 356.9 | Unspecified hereditary and idiopathic peripheral neuropathy |
| 09 | 357.2 | Polyneuropathy in diabetes |
| 09 | 357.4 | Polyneuropathy in other diseases classified elsewhere |
| 10 | E08.40 | Diabetes mellitus due to underlying condition with diabetic neuropathy, unspecified |
| 10 | E08.4 |  |
| 10 | E08.42 | Diabetes mellitus due to underlying condition with diabetic polyneuropathy |
| 10 | E08.49 | Diabetes mellitus due to underlying condition with other diabetic neurological complication |
| 10 | E09.4 |  |
| 10 | E09.40 | Drug or chemical induced diabetes mellitus with neurological complications with diabetic neuropathy, unspecified |
| 10 | E09.42 | Drug or chemical induced diabetes mellitus with neurological complications with diabetic polyneuropathy |
| 10 | E09.49 | Drug or chemical induced diabetes mellitus with neurological complications with other diabetic neurological complication |
| 10 | E10.4 |  |
| 10 | E10.40 | Type 1 diabetes mellitus with diabetic neuropathy, unspecified |
| 10 | E10.41 | Type 1 diabetes mellitus with diabetic mononeuropathy |
| 10 | E10.42 | Type 1 diabetes mellitus with diabetic polyneuropathy |
| 10 | E10.49 | Type 1 diabetes mellitus with other diabetic neurological complication |
| 10 | E11.4 |  |
| 10 | E11.40 | Type 2 diabetes mellitus with diabetic neuropathy, unspecified |
| 10 | E11.41 | Type 2 diabetes mellitus with diabetic mononeuropathy |
| 10 | E11.42 | Type 2 diabetes mellitus with diabetic polyneuropathy |
| 10 | E11.49 | Type 2 diabetes mellitus with other diabetic neurological complication |
| 10 | E13.4 |  |
| 10 | E13.40 | Other specified diabetes mellitus with diabetic neuropathy, unspecified |
| 10 | E13.41 | Other specified diabetes mellitus with diabetic mononeuropathy |
| 10 | E13.42 | Other specified diabetes mellitus with diabetic polyneuropathy |
| 10 | E13.49 | Other specified diabetes mellitus with other diabetic neurological complication |
| 10 | G60.3 | Idiopathic progressive |
| 10 | G60.8 | Other hereditary and idiopathic |
| 10 | G60.9 | hereditary and idiopathic, unspecified |
| 10 | G62.9 | Polyneuropathy, unspecified |
| 10 | G63 | Polyneuropathy in disease classified elsewhere |
