# Supplementary material for: Estimating the Risk of Lower Extremity Complications in Adults Newly Diagnosed With Diabetic Polyneuropathy: Retrospective Cohort Study
Source: JMIR Diabetes. 2025 May 29;10:e60141. doi: 10.2196/60141 (PMC12140504; doi:10.2196/60141)
Supplement: Multimedia Appendix 2 [file diabetes-v10-e60141-s002.docx]

Appendix 2. Procedures for identifying onset of lower extremity complications among adults newly diagnosed with diabetic polyneuropathy.

| **Type of Event^a^** | **At least one inpatient or outpatient diagnosis with the following ICD-9 or ICD-10 codes** |  | **At least 1 Procedure Code** | |  | **Prescription Drugs** |
| --- | --- | --- | --- | --- | --- | --- |
| *Foot ulcer* | 707.14, 707.15, E10.621,E11.621, E13.621, I70.234, I70.235, I70.244, I70.245, L97.401, L97.402, L97.403, L97.404, L97.409, L97.411, L97.412,  L97.413, L97.414, L97.419, L97.421,  L97.422, L97.423, L97.424, L97.429,  L97.501, L97.502, L97.503, L97.504,  L97.509, L97.511, L97.512, L97.513,  L97.514, L97.519, L97.521, L97.522,  L97.523, L97.524, L97.529 | OR | 11042, 11043, 11044, 11045, 11046, 11047 |  | |  |
| *Osteomyelitis* | 730.07, 730.17, 730.27, 730.97, M86.171, M86.172, M86.179, M86.271, M86.272, M86.279, M86.471, M86.472, M86.479, M86.671, M86.672, M86.679, M86.8X7, M86.9 |  |  | AND^b^ | | cefazolin, cefepime,  ceftazidime, ceftriaxone, ciprofloxacin, daptomycin, ertapenem, levofloxacin, linezolid  nafcillin, oxacillin, vancomycin, clindamycin, cefalexin, amoxicillin, doxycycline  trimethoprim-sulfamethoxazole, cefoxitin, ampicillin, moxifloxacin, tigecycline(Bs), imipenem/cilastatin, piperacillin |
| *Gangrene* | 040.0, 785.4, A48.0, E10.52, E11.52, E13.52, I96 |  |  |  | |  |
| *Non-traumatic Lower Extremity Amputation* | V49.70, V49.71, V49.72, V49.73, V49.74, V49.75, V49.76, V49.77, V52.1, 84.10, 84.11, 84.12, 84.13, 84.14, 84.15, 84.16, 84.17,  84.18, Z89.411, Z89.412, Z89.419, Z89.421, Z89.422, Z89.429, Z89.431, Z89.432, Z89.439, Z89.441, Z89.442, Z89.449, Z89.511, Z89.512, Z89.519, Z89.611, Z89.612, Z89.619, Z89.621, Z89.622, Z89.629, Z89.9, 0Y6F0ZZ, 0Y6G0ZZ, 0Y6H0Z1, 0Y6H0Z2, 0Y6H0Z3, 0Y6J0Z1, 0Y6J0Z2, 0Y6J0Z3, 0Y6M0Z0, 0Y6M0Z4, 0Y6M0Z5, 0Y6M0Z6, 0Y6M0Z7, 0Y6M0Z8, 0Y6M0Z9, 0Y6M0ZB, 0Y6M0ZC, 0Y6M0ZD, 0Y6M0ZF, 0Y6N0Z0, 0Y6N0Z4, 0Y6N0Z5, 0Y6N0Z6, 0Y6N0Z7, 0Y6N0Z8, 0Y6N0Z9, 0Y6N0ZB, 0Y6N0ZC, 0Y6N0ZD, 0Y6N0ZF, 0Y6P0Z0, 0Y6P0Z1, 0Y6P0Z2, 0Y6P0Z3, 0Y6Q0Z0, 0Y6Q0Z1, 0Y6Q0Z2, 0Y6Q0Z3, 0Y6R0Z0, 0Y6R0Z1, 0Y6R0Z2, 0Y6R0Z3, 0Y6S0Z0, 0Y6S0Z1, 0Y6S0Z2, 0Y6S0Z3, 0Y6T0Z0, 0Y6T0Z1, 0Y6T0Z2, 0Y6T0Z3, 0Y6U0Z0, 0Y6U0Z1, 0Y6U0Z2, 0Y6U0Z3, 0Y6V0Z0, 0Y6V0Z1, 0Y6V0Z2, 0Y6V0Z3, 0Y6W0Z0, 0Y6W0Z1, 0Y6W0Z2, 0Y6W0Z3, 0Y6X0Z0, 0Y6X0Z1, 0Y6X0Z2, 0Y6X0Z3, 0Y6Y0Z0, 0Y6Y0Z1, 0Y6Y0Z2, 0Y6Y0Z3 | AND^c^ | 27290, 27295, 27598, 27880, 27881, 27882, 27884, 27886, 27888, 27889, 28800, 28805, 28810, 28820, 28825 |  | |  |

a. New event is defined as no evidence of an event during the prior 100 days for foot ulcer, 120 days for osteomyelitis, and 160 days for gangrene or lower extremity amputation.

b. For outpatient diagnoses, at least one medication order for an antibiotic within 7 days before or 14 days after the diagnosis is also required.

c. For inpatient diagnosis, procedure code is the primary indication of an event, followed by diagnosis code. For outpatient diagnosis, at least one procedure code was required to occur within 24 hours.
