# Supplementary material for: Estimating the Risk of Lower Extremity Complications in Adults Newly Diagnosed With Diabetic Polyneuropathy: Retrospective Cohort Study
Source: JMIR Diabetes. 2025 May 29;10:e60141. doi: 10.2196/60141 (PMC12140504; doi:10.2196/60141)
Supplement: Multimedia Appendix 3 [file diabetes-v10-e60141-s003.docx]

*Appendix 3. Study cohort description*

| **Independent factor** | **Level** | **Training (N=38,568) n(%)** | **Validation (N=9,641) n(%)** | **Entire**  **(N=48,209)**  **n(%)** |
| --- | --- | --- | --- | --- |
| Patient age at time of DPN diagnosis | Mean (SD) | 64.38 (12.40) | 64.21 (12.43) | 64.34 (12.41) |
| Sex |  |  |  |  |
|  | 1: Female | 17924 (46.47) | 4455 (46.21) | 22379 (46.42) |
|  | 2: Male | 20643 (53.52) | 5185 (53.78) | 25828 (53.58) |
|  | 9: Unknown | 1 (0.00) | 1 (0.01) | 2 (0.00) |
| Race/Ethnicity |  |  |  |  |
|  | 1: Asian | 4930 (12.78) | 1165 (12.08) | 6095 (12.64) |
|  | 2: Black | 4137 (10.73) | 1084 (11.24) | 5221 (10.83) |
|  | 3: Native Hawaiian, Pacific Islander, Native American | 636 (1.65) | 161 (1.67) | 797 (1.65) |
|  | 4: Hispanic | 7743 (20.08) | 1965 (20.38) | 9708 (20.14) |
|  | 5: White | 20102 (52.12) | 5008 (51.94) | 25110 (52.09) |
|  | 9: Unknown | 1020 (2.64) | 258 (2.68) | 1278 (2.65) |
| Year of DPN recognition |  |  |  |  |
|  | 2012 | 7150 (18.54) | 1791 (18.58) | 8941 (18.55) |
|  | 2013 | 8949 (23.20) | 2258 (23.42) | 11207 (23.25) |
|  | 2014 | 8394 (21.76) | 2083 (21.61) | 10477 (21.73) |
|  | 2015 | 7289 (18.90) | 1836 (19.04) | 9125 (18.93) |
|  | 2016 | 6786 (17.59) | 1673 (17.35) | 8459 (17.55) |
| Number of days after diagnosis of DPN | Mean (SD) | 549.36 (239.23) | 549.82 (238.87) | 549.45 (239.15) |
| Reason for end of follow up |  |  |  |  |
|  | 1:Event | 1862 (4.83) | 465 (4.82) | 2327 (4.83) |
|  | 2:Admin End | 32837 (85.14) | 8209 (85.15) | 41046 (85.14) |
|  | 3:Disenroll | 2284 (5.92) | 571 (5.92) | 2855 (5.92) |
|  | 4:Death | 1585 (4.11) | 396 (4.11) | 1981 (4.11) |
| COPS2^a^ at baseline calculated using DX records within 12 months prior to the DPN diagnosis [Range: 0-700; values >300 are uncommon] | Mean (SD) | 36.95 (30.98) | 36.73 (31.30) | 36.91 (31.05) |
| Event history within 24 months prior to DPN diagnosis | Mean (SD) | 0.08 (0.40) | 0.08 (0.41) | 0.08 (0.40) |
| Number of insulin prescription fills within 24 months prior to the DPN DX | Mean (SD) | 2.67 (5.09) | 2.61 (5.03) | 2.65 (5.08) |
| Any lab test results for HbA1C in prior 24 months |  |  |  |  |
|  | 0: No | 1481 (3.84) | 367 (3.81) | 1848 (3.83) |
|  | 1: Yes | 37087 (96.16) | 9274 (96.19) | 46361 (96.17) |
| Number of HbA1C lab tests within 24 months prior to the DPN diagnosis | Mean (SD) | 3.76 (2.11) | 3.74 (2.10) | 3.75 (2.10) |
| Mean HbA1c assessed within 24 months prior to the DPN diagnosis | Mean (SD) | 7.85 (1.69) | 7.84 (1.70) | 7.85 (1.70) |
| Imputation flag for mean A1c (missing values) |  |  |  |  |
|  | 0: No | 37087 (96.16) | 9274 (96.19) | 46361 (96.17) |
|  | 1: Yes | 1481 (3.84) | 367 (3.81) | 1848 (3.83) |
| Any HDL measurements in prior 24 months |  |  |  |  |
|  | 0: No | 3303 (8.56) | 796 (8.26) | 4099 (8.50) |
|  | 1: Yes | 35265 (91.44) | 8845 (91.74) | 44110 (91.50) |
| Mean Lab test results for high-density lipoprotein recorded within 24 months prior to the DPN diagnosis | Mean (SD) | 46.09 (11.75) | 45.88 (11.56) | 46.05 (11.72) |
| Imputation flag for mean HDL (missing values) |  |  |  |  |
|  | 0: No | 35265 (91.44) | 8845 (91.74) | 44110 (91.50) |
|  | 1: Yes | 3303 (8.56) | 796 (8.26) | 4099 (8.50) |
| Any TCL measurements in prior 24 months |  |  |  |  |
|  | 0: No | 3131 (8.12) | 757 (7.85) | 3888 (8.06) |
|  | 1: Yes | 35437 (91.88) | 8884 (92.15) | 44321 (91.94) |
| Mean Lab test results for total cholesterol recorded within 24 months prior to the DPN diagnosis | Mean (SD) | 167.93 (39.22) | 167.24 (38.04) | 167.79 (38.99) |
| Imputation flag for mean TCL (missing values) |  |  |  |  |
|  | 0: No | 35437 (91.88) | 8884 (92.15) | 44321 (91.94) |
|  | 1: Yes | 3131 (8.12) | 757 (7.85) | 3888 (8.06) |
| Any TRIG measurements in prior 24 months |  |  |  |  |
|  | 0: No | 8189 (21.23) | 2009 (20.84) | 10198 (21.15) |
|  | 1: Yes | 30379 (78.77) | 7632 (79.16) | 38011 (78.85) |
| Mean lab test results for triglycerides recorded within 24 months prior to the DPN diagnosis | Mean (SD) | 179.12 (146.33) | 176.16 (124.58) | 178.53 (142.25) |
| Imputation flag for mean TRIG (missing values) |  |  |  |  |
|  | 0: No | 30379 (78.77) | 7632 (79.16) | 38011 (78.85) |
|  | 1: Yes | 8189 (21.23) | 2009 (20.84) | 10198 (21.15) |
| Any body mass index (BMI) measurements in prior 24 months |  |  |  |  |
|  | 0: No | 613 (1.59) | 186 (1.93) | 799 (1.66) |
|  | 1: Yes | 37955 (98.41) | 9455 (98.07) | 47410 (98.34) |
| Mean BMI recorded within 24 months prior to the DPN diagnosis | Mean (SD) | 33.04 (90.35) | 32.34 (10.05) | 32.90 (80.94) |
| Imputation flag for mean BMI (missing values) |  |  |  |  |
|  | 0: No | 37955 (98.41) | 9455 (98.07) | 47410 (98.34) |
|  | 1: Yes | 613 (1.59) | 186 (1.93) | 799 (1.66) |
| Any systolic blood pressure (SBP) measurements in prior 24 months |  |  |  |  |
|  | 0: No | 381 (0.99) | 110 (1.14) | 491 (1.02) |
|  | 1: Yes | 38187 (99.01) | 9531 (98.86) | 47718 (98.98) |
| Mean SBP recorded within 24 months prior to the DPN diagnosis | Mean (SD) | 130.66 (11.85) | 130.52 (11.71) | 130.63 (11.82) |
| Imputation flag for mean SBP (missing values) |  |  |  |  |
|  | 0: No | 38187 (99.01) | 9531 (98.86) | 47718 (98.98) |
|  | 1: Yes | 381 (0.99) | 110 (1.14) | 491 (1.02) |
| Any diastolic blood pressure (DBP) measurements in prior 24 months |  |  |  |  |
|  | 0: No | 381 (0.99) | 110 (1.14) | 491 (1.02) |
|  | 1: Yes | 38187 (99.01) | 9531 (98.86) | 47718 (98.98) |
| Mean DBP recorded within 24 months prior to the DPN Diagnosis | Mean (SD) | 72.50 (7.98) | 72.62 (8.01) | 72.52 (7.98) |
| Imputation flag for mean DBP (missing values) |  |  |  |  |
|  | 0: No | 38187 (99.01) | 9531 (98.86) | 47718 (98.98) |
|  | 1: Yes | 381 (0.99) | 110 (1.14) | 491 (1.02) |
| Any dispensing of opioids in prior 24 months |  |  |  |  |
|  | 0: No | 18994 (49.25) | 4762 (49.39) | 23756 (49.28) |
|  | 1: Yes | 19574 (50.75) | 4879 (50.61) | 24453 (50.72) |
| Any dispensing of dyslipidemia drugs in prior 24 months |  |  |  |  |
|  | 0: No | 7528 (19.52) | 1884 (19.54) | 9412 (19.52) |
|  | 1: Yes | 31040 (80.48) | 7757 (80.46) | 38797 (80.48) |
| Any dispensing of antihypertensive drugs in prior 24 months |  |  |  |  |
|  | 0: No | 5698 (14.77) | 1550 (16.08) | 7248 (15.03) |
|  | 1: Yes | 32870 (85.23) | 8091 (83.92) | 40961 (84.97) |
| Any dispensing of antidiabetic drugs in prior 24 months |  |  |  |  |
|  | 0: No | 8277 (21.46) | 2066 (21.43) | 10343 (21.45) |
|  | 1: Yes | 30291 (78.54) | 7575 (78.57) | 37866 (78.55) |
| Any dispensing of cardiovascular (CVD) drugs in prior 24 months |  |  |  |  |
|  | 0: No | 3057 (7.93) | 786 (8.15) | 3843 (7.97) |
|  | 1: Yes | 35511 (92.07) | 8855 (91.85) | 44366 (92.03) |
| Any diagnoses of peripheral artery disease in prior 24 months |  |  |  |  |
|  | 0: No | 37192 (96.43) | 9283 (96.29) | 46475 (96.40) |
|  | 1: Yes | 1376 (3.57) | 358 (3.71) | 1734 (3.60) |
| Any diagnoses of cellulitis in prior 24 months |  |  |  |  |
|  | 0: No | 37019 (95.98) | 9251 (95.95) | 46270 (95.98) |
|  | 1: Yes | 1549 (4.02) | 390 (4.05) | 1939 (4.02) |
| Any diagnoses of chronic pain in prior 24 months |  |  |  |  |
|  | 0: No | 10597 (27.48) | 2678 (27.78) | 13275 (27.54) |
|  | 1: Yes | 27971 (72.52) | 6963 (72.22) | 34934 (72.46) |
| Any diagnoses of atrial flutter or atrial fibrillation in prior 24 months |  |  |  |  |
|  | 0: No | 35205 (91.28) | 8807 (91.35) | 44012 (91.29) |
|  | 1: Yes | 3363 (8.72) | 834 (8.65) | 4197 (8.71) |
| Any diagnoses of heart failure or cardiovascular disease in prior 24 months |  |  |  |  |
|  | 0: No | 32948 (85.43) | 8282 (85.90) | 41230 (85.52) |
|  | 1: Yes | 5620 (14.57) | 1359 (14.10) | 6979 (14.48) |
| Any diagnoses of chronic kidney disease in prior 24 months |  |  |  |  |
|  | 0: No | 25487 (66.08) | 6460 (67.01) | 31947 (66.27) |
|  | 1: Yes | 13081 (33.92) | 3181 (32.99) | 16262 (33.73) |
| Any diagnoses of rheumatoid arthritis in prior 24 months |  |  |  |  |
|  | 0: No | 36747 (95.28) | 9149 (94.90) | 45896 (95.20) |
|  | 1: Yes | 1821 (4.72) | 492 (5.10) | 2313 (4.80) |
| Any diagnoses of diabetic retinopathy or macular edema in prior 24 months |  |  |  |  |
|  | 0: No | 30297 (78.55) | 7590 (78.73) | 37887 (78.59) |
|  | 1: Yes | 8271 (21.45) | 2051 (21.27) | 10322 (21.41) |
| Any diagnoses of fall-related injuries in prior 24 months |  |  |  |  |
|  | 0: No | 37147 (96.32) | 9290 (96.36) | 46437 (96.32) |
|  | 1: Yes | 1421 (3.68) | 351 (3.64) | 1772 (3.68) |
| Any diagnoses of ischemic and hemorrhagic strokes in prior 24 months |  |  |  |  |
|  | 0: No | 36616 (94.94) | 9178 (95.20) | 45794 (94.99) |
|  | 1: Yes | 1952 (5.06) | 463 (4.80) | 2415 (5.01) |
| Any diagnoses of sleep apnea in prior 24 months |  |  |  |  |
|  | 0: No | 33734 (87.47) | 8442 (87.56) | 42176 (87.49) |
|  | 1: Yes | 4834 (12.53) | 1199 (12.44) | 6033 (12.51) |
| Any diagnoses of other neuropathy in prior 24 months |  |  |  |  |
|  | 0: No | 30502 (79.09) | 7643 (79.28) | 38145 (79.12) |
|  | 1: Yes | 8066 (20.91) | 1998 (20.72) | 10064 (20.88) |
| Any diagnoses of Charcot-foot disorder in prior 24 months |  |  |  |  |
|  | 0: No | 38556 (99.97) | 9638 (99.97) | 48194 (99.97) |
|  | 1: Yes | 12 (0.03) | 3 (0.03) | 15 (0.03) |
| Any treatment of hyperbaric oxygen therapy in prior 24 months |  |  |  |  |
|  | 0: No | 38559 (99.98) | 9638 (99.97) | 48197 (99.98) |
|  | 1: Yes | 9 (0.02) | 3 (0.03) | 12 (0.02) |
| Types of diabetes diagnosed in prior 24 months |  |  |  |  |
|  | 1: Type 1 DM | 999 (2.59) | 239 (2.48) | 1238 (2.57) |
|  | 2: Type 2 DM | 35758 (92.71) | 8947 (92.80) | 44705 (92.73) |
|  | 3: Unknown DM type | 1811 (4.70) | 455 (4.72) | 2266 (4.70) |
| Most recent smoking status at baseline |  |  |  |  |
|  | 0: Nonsmoker | 20327 (52.70) | 5004 (51.90) | 25331 (52.54) |
|  | 1: Former | 13455 (34.89) | 3396 (35.22) | 16851 (34.95) |
|  | 2: Smoker | 2979 (7.72) | 754 (7.82) | 3733 (7.74) |
|  | 9: Unknown | 1807 (4.69) | 487 (5.05) | 2294 (4.76) |
| Alcohol consumption at baseline |  |  |  |  |
|  | 0: No | 18915 (49.04) | 4690 (48.65) | 23605 (48.96) |
|  | 1: Yes | 11269 (29.22) | 2873 (29.80) | 14142 (29.33) |
|  | 9: NA/Unknown | 8384 (21.74) | 2078 (21.55) | 10462 (21.70) |
