# Supplementary material for: Estimating the Risk of Lower Extremity Complications in Adults Newly Diagnosed With Diabetic Polyneuropathy: Retrospective Cohort Study
Source: JMIR Diabetes. 2025 May 29;10:e60141. doi: 10.2196/60141 (PMC12140504; doi:10.2196/60141)
Supplement: Multimedia Appendix 4 [file diabetes-v10-e60141-s004.docx]

**Multimedia Appendix 4.** **Detailed description of algorithm development.**

Following the general approach of Polley and van der Laan^34^, we used Super Learning to construct a single point estimator of the vector of the discrete-time conditional hazards, i.e., h(t,W) = P(Y(t)=1|Y(t-1)=0, W) for t=1,2,… where Y(t) represent the outcome at time t and W is the vector of candidate covariates. Follow-up time t was coarsened using the quarter (91-day interval) as the unit of time. We then mapped this estimator into a predictor of cumulative incidence over 8 quarters calculated as follows $P\left( Y\left( 8 \right)=1 | W \right)=1- \prod_{t=1}^{8} (1-h(t,W))$. We considered 22 candidate hazard predictors defined by a naïve mean model, main-term logistic regressions, penalized regressions (elastic net, lasso, ridge), random forests (trees=50, 250, 1000), and gradient boosting (trees=25, 50, 100). Each candidate hazard predictor considered ($h_{n}(t,W)$) was defined in two ways: as a time-stratified estimator (i.e., $h_{n}(t,W)$=($h_{n,1}(t,W)$,…,$h_{n,8}(t,W)$)) and as a pooled estimator across all 8 quarters of data. For example, the simplest candidate predictor we considered $h_{n}(t,W)$) based on the naïve mean approach was defined as the vector of 8 separate regression fits that were each derived by fitting the naïve mean approach for predicting the hazard at quarter t using data from only quarter t. In contrast, in the pooled approach, we fit the naive mean approach once using data pooled over all quarters t=1,...,8. Implementation was based on the sl3 R package^35^ using 10-fold cross-validation and the L2 loss function.
