# Supplementary material for: Estimating the Risk of Lower Extremity Complications in Adults Newly Diagnosed With Diabetic Polyneuropathy: Retrospective Cohort Study
Source: JMIR Diabetes. 2025 May 29;10:e60141. doi: 10.2196/60141 (PMC12140504; doi:10.2196/60141)
Supplement: Multimedia Appendix 5 [file diabetes-v10-e60141-s005.docx]

Appendix 5. Comparison of predictor characteristics by event type.

| **Independent Factor** | **Level** | **Adverse Outcome N=2,327 (4.83%)**  **n(%)** | **No Adverse Outcome N=25,180**  **(52.2%) n(%)** | **Censored N=20,702 (42.9%) n(%)** |
| --- | --- | --- | --- | --- |
| Patient age at time of diabetic polyneuropathy (DPN) diagnosis (Dx) | Mean (SD) | 63.83 (12.74) | 64.08 (11.92) | 64.72 (12.93) |
| Sex |  |  |  |  |
|  | 1: Female | 740 (31.80) | 12003 (47.67) | 9636 (46.55) |
|  | 2: Male | 1587 (68.20) | 13175 (52.32) | 11066 (53.45) |
|  | 9: Unknown | 0 (0.00) | 2 (0.00) | 0 (0.00) |
| Race/Ethnicity |  |  |  |  |
|  | 1: Asian | 138 (5.93) | 3285 (13.05) | 2672 (12.91) |
|  | 2: Black | 242 (10.40) | 2765 (10.98) | 2214 (10.69) |
|  | 3: Native Hawaiian/  Pacific Islander/  Native American | 34 (1.46) | 413 (1.64) | 350 (1.69) |
|  | 4: HISPANIC | 430 (18.48) | 4896 (19.44) | 4382 (21.17) |
|  | 5: WHITE | 1435 (61.67) | 13239 (52.58) | 10436 (50.41) |
|  | 9: Unknown | 48 (2.06) | 582 (2.31) | 648 (3.13) |
| Year of DPN recognition |  |  |  |  |
|  | 2012 | 498 (21.40) | 7360 (29.23) | 1083 (5.23) |
|  | 2013 | 633 (27.20) | 9183 (36.47) | 1391 (6.72) |
|  | 2014 | 590 (25.35) | 8636 (34.30) | 1251 (6.04) |
|  | 2015 | 422 (18.13) | 1 (0.00) | 8702 (42.03) |
|  | 2016 | 184 (7.91) | 8275 (39.97) | . (.) |
| Number of days after DPN Dx | Mean (SD) | 274.21 (208.33) | 731.00 (0.00) | 359.57 (208.93) |
| COPS2 at baseline calculated using Dx records within 12 months prior to the DPN diagnosis [Range: 0-315] | Mean (SD) | 57.55 (40.17) | 32.62 (26.08) | 39.80 (34.02) |
| Event history within 24 months prior to DPN diagnosis | Mean (SD) | 0.66 (1.04) | 0.04 (0.26) | 0.06 (0.37) |
| Number of insulin prescription fills within 24 months prior to the DPN Dx | Mean (SD) | 3.88 (5.76) | 2.57 (5.10) | 2.62 (4.95) |
| Any lab test results for Hemoglobin A1_C_ (HbA1c) in prior 24 months |  |  |  |  |
|  | 0: No | 165 (7.09) | 1100 (4.37) | 583 (2.82) |
|  | 1: Yes | 2162 (92.91) | 24080 (95.63) | 20119 (97.18) |
| Number of HbA1c lab tests within 24 months prior to the DPN diagnosis | Mean (SD) | 3.53 (2.29) | 3.72 (2.04) | 3.82 (2.15) |
| Mean HbA1c assessed within 24 months prior to the DPN diagnosis | Mean (SD) | 8.50 (2.05) | 7.76 (1.62) | 7.89 (1.72) |
| Imputation flag for mean HbA1c (missing values) |  |  |  |  |
|  | 0: No | 2162 (92.91) | 24080 (95.63) | 20119 (97.18) |
|  | 1: Yes | 165 (7.09) | 1100 (4.37) | 583 (2.82) |
| Any high-density lipoprotein (HDL) measurements in prior 24 months |  |  |  |  |
|  | 0: No | 279 (11.99) | 1163 (4.62) | 2657 (12.83) |
|  | 1: Yes | 2048 (88.01) | 24017 (95.38) | 18045 (87.17) |
| Mean lab test results for high-density lipoprotein recorded within 24 months prior to the DPN diagnosis | Mean (SD) | 44.10 (11.83) | 46.22 (11.71) | 46.06 (11.69) |
| Imputation flag for mean HDL (missing values) |  |  |  |  |
|  | 0: No | 2048 (88.01) | 24017 (95.38) | 18045 (87.17) |
|  | 1: Yes | 279 (11.99) | 1163 (4.62) | 2657 (12.83) |
| Any total cholesterol (TCL) measurements in prior 24 months |  |  |  |  |
|  | 0: No | 262 (11.26) | 1122 (4.46) | 2504 (12.10) |
|  | 1: Yes | 2065 (88.74) | 24058 (95.54) | 18198 (87.90) |
| Mean lab test results for total cholesterol recorded within 24 months prior to the DPN diagnosis | Mean (SD) | 166.97 (42.09) | 168.25 (37.28) | 167.33 (40.62) |
| Imputation flag for mean triglycerides (missing values) |  |  |  |  |
|  | 0: No | 2065 (88.74) | 24058 (95.54) | 18198 (87.90) |
|  | 1: Yes | 262 (11.26) | 1122 (4.46) | 2504 (12.10) |
| Any triglycerides measurements in prior 24 months |  |  |  |  |
|  | 0: No | 550 (23.64) | 3680 (14.61) | 5968 (28.83) |
|  | 1: Yes | 1777 (76.36) | 21500 (85.39) | 14734 (71.17) |
| Mean lab test results for triglycerides recorded within 24 months prior to the DPN diagnosis | Mean (SD) | 189.43 (154.84) | 175.94 (130.10) | 180.45 (154.34) |
| Imputation flag for mean triglycerides (missing values) |  |  |  |  |
|  | 0: No | 1777 (76.36) | 21500 (85.39) | 14734 (71.17) |
|  | 1: Yes | 550 (23.64) | 3680 (14.61) | 5968 (28.83) |
| Any body mass index (BMI) measurements in prior 24 months |  |  |  |  |
|  | 0: No | 69 (2.97) | 321 (1.27) | 409 (1.98) |
|  | 1: Yes | 2258 (97.03) | 24859 (98.73) | 20293 (98.02) |
| Mean BMI recorded within 24 months prior to the DPN diagnosis | Mean (SD) | 33.60 (29.15) | 33.35 (110.75) | 32.28 (15.51) |
| Imputation flag for mean BMI (missing values) |  |  |  |  |
|  | 0: No | 2258 (97.03) | 24859 (98.73) | 20293 (98.02) |
|  | 1: Yes | 69 (2.97) | 321 (1.27) | 409 (1.98) |
| Any systolic blood pressure (SBP) measurements in prior 24 months |  |  |  |  |
|  | 0: No | 36 (1.55) | 153 (0.61) | 302 (1.46) |
|  | 1: Yes | 2291 (98.45) | 25027 (99.39) | 20400 (98.54) |
| Mean SBP recorded within 24 months prior to the DPN diagnosis | Mean (SD) | 132.85 (13.29) | 130.30 (11.62) | 130.79 (11.86) |
| Imputation flag for mean SBP (missing values) |  |  |  |  |
|  | 0: No | 2291 (98.45) | 25027 (99.39) | 20400 (98.54) |
|  | 1: Yes | 36 (1.55) | 153 (0.61) | 302 (1.46) |
| Any diastolic blood pressure (DBP) measurements in prior 24 months |  |  |  |  |
|  | 0: No | 36 (1.55) | 153 (0.61) | 302 (1.46) |
|  | 1: Yes | 2291 (98.45) | 25027 (99.39) | 20400 (98.54) |
| Mean DBP recorded within 24 months prior to the DPN Dx | Mean (SD) | 72.55 (8.62) | 72.67 (7.85) | 72.34 (8.07) |
| Imputation flag for mean DBP (missing values) |  |  |  |  |
|  | 0: No | 2291 (98.45) | 25027 (99.39) | 20400 (98.54) |
|  | 1: Yes | 36 (1.55) | 153 (0.61) | 302 (1.46) |
| Any dispensing of opioids in prior 24 months |  |  |  |  |
|  | 0: No | 1009 (43.36) | 12570 (49.92) | 10177 (49.16) |
|  | 1: Yes | 1318 (56.64) | 12610 (50.08) | 10525 (50.84) |
| Any dispensing of dyslipidemia drugs in prior 24 months |  |  |  |  |
|  | 0: No | 585 (25.14) | 4819 (19.14) | 4008 (19.36) |
|  | 1: Yes | 1742 (74.86) | 20361 (80.86) | 16694 (80.64) |
| Any dispensing of antihypertensive drugs in prior 24 months |  |  |  |  |
|  | 0: No | 305 (13.11) | 3715 (14.75) | 3228 (15.59) |
|  | 1: Yes | 2022 (86.89) | 21465 (85.25) | 17474 (84.41) |
| Any dispensing of antidiabetic drugs in prior 24 months |  |  |  |  |
|  | 0: No | 421 (18.09) | 5651 (22.44) | 4271 (20.63) |
|  | 1: Yes | 1906 (81.91) | 19529 (77.56) | 16431 (79.37) |
| Any dispensing of cardiovascular drugs in prior 24 months |  |  |  |  |
|  | 0: No | 217 (9.33) | 1945 (7.72) | 1681 (8.12) |
|  | 1: Yes | 2110 (90.67) | 23235 (92.28) | 19021 (91.88) |
| Any diagnoses of peripheral artery disease in prior 24 months |  |  |  |  |
|  | 0: No | 2062 (88.61) | 24520 (97.38) | 19893 (96.09) |
|  | 1: Yes | 265 (11.39) | 660 (2.62) | 809 (3.91) |
| Any diagnoses of cellulitis in prior 24 months |  |  |  |  |
|  | 0: No | 1886 (81.05) | 24472 (97.19) | 19912 (96.18) |
|  | 1: Yes | 441 (18.95) | 708 (2.81) | 790 (3.82) |
| Any diagnoses of chronic pain in prior 24 months |  |  |  |  |
|  | 0: No | 740 (31.80) | 7164 (28.45) | 5371 (25.94) |
|  | 1: Yes | 1587 (68.20) | 18016 (71.55) | 15331 (74.06) |
| Any diagnoses of atrial flutter or atrial fibrillation in prior 24 months |  |  |  |  |
|  | 0: No | 2013 (86.51) | 23324 (92.63) | 18675 (90.21) |
|  | 1: Yes | 314 (13.49) | 1856 (7.37) | 2027 (9.79) |
| Any diagnoses of heart failure or cardiovascular disease in prior 24 months |  |  |  |  |
|  | 0: No | 1756 (75.46) | 22204 (88.18) | 17270 (83.42) |
|  | 1: Yes | 571 (24.54) | 2976 (11.82) | 3432 (16.58) |
| Any diagnoses of chronic kidney disease in prior 24 months |  |  |  |  |
|  | 0: No | 1254 (53.89) | 17049 (67.71) | 13644 (65.91) |
|  | 1: Yes | 1073 (46.11) | 8131 (32.29) | 7058 (34.09) |
| Any diagnoses of rheumatoid arthritis in prior 24 months |  |  |  |  |
|  | 0: No | 2218 (95.32) | 24094 (95.69) | 19584 (94.60) |
|  | 1: Yes | 109 (4.68) | 1086 (4.31) | 1118 (5.40) |
| Any diagnoses of diabetic retinopathy or macular edema in prior 24 months |  |  |  |  |
|  | 0: No | 1414 (60.76) | 20440 (81.18) | 16033 (77.45) |
|  | 1: Yes | 913 (39.24) | 4740 (18.82) | 4669 (22.55) |
| Any diagnoses of fall-related Injuries in prior 24 months |  |  |  |  |
|  | 0: No | 2197 (94.41) | 24397 (96.89) | 19843 (95.85) |
|  | 1: Yes | 130 (5.59) | 783 (3.11) | 859 (4.15) |
| Any diagnoses of ischemic and hemorrhagic strokes in prior 24 months |  |  |  |  |
|  | 0: No | 2169 (93.21) | 24182 (96.04) | 19443 (93.92) |
|  | 1: Yes | 158 (6.79) | 998 (3.96) | 1259 (6.08) |
| Any diagnoses of sleep apnea in prior 24 months |  |  |  |  |
|  | 0: No | 2030 (87.24) | 22193 (88.14) | 17953 (86.72) |
|  | 1: Yes | 297 (12.76) | 2987 (11.86) | 2749 (13.28) |
| Any diagnoses of other neuropathy in prior 24 months |  |  |  |  |
|  | 0: No | 1787 (76.79) | 21141 (83.96) | 15217 (73.50) |
|  | 1: Yes | 540 (23.21) | 4039 (16.04) | 5485 (26.50) |
| Any diagnoses of Charcot-Foot disorder in prior 24 months |  |  |  |  |
|  | 0: No | 2325 (99.91) | 25174 (99.98) | 20695 (99.97) |
|  | 1: Yes | 2 (0.09) | 6 (0.02) | 7 (0.03) |
| Any treatment of hyperbaric oxygen therapy in prior 24 months |  |  |  |  |
|  | 0: No | 2326 (99.96) | 25178 (99.99) | 20693 (99.96) |
|  | 1: Yes | 1 (0.04) | 2 (0.01) | 9 (0.04) |
| Types of diabetes diagnoses in prior 24 months |  |  |  |  |
|  | 1: Type 1 DM | 77 (3.31) | 608 (2.41) | 553 (2.67) |
|  | 2: Type 2 DM | 2144 (92.14) | 22966 (91.21) | 19595 (94.65) |
|  | 3: Unknown DM type | 106 (4.56) | 1606 (6.38) | 554 (2.68) |
| Most recent smoking status at baseline |  |  |  |  |
|  | 0: Nonsmoker | 1147 (49.29) | 13349 (53.01) | 10835 (52.34) |
|  | 1: Former | 798 (34.29) | 8662 (34.40) | 7391 (35.70) |
|  | 2: Smoker | 211 (9.07) | 1918 (7.62) | 1604 (7.75) |
|  | 9: Unknown | 171 (7.35) | 1251 (4.97) | 872 (4.21) |
| Alcohol consumption at baseline |  |  |  |  |
|  | 0: No | 1115 (47.92) | 12047 (47.84) | 10443 (50.44) |
|  | 1: Yes | 659 (28.32) | 7121 (28.28) | 6362 (30.73) |
|  | 9: NA/Unknown | 553 (23.76) | 6012 (23.88) | 3897 (18.82) |
