# Supplementary material for: Estimating the Risk of Lower Extremity Complications in Adults Newly Diagnosed With Diabetic Polyneuropathy: Retrospective Cohort Study
Source: JMIR Diabetes. 2025 May 29;10:e60141. doi: 10.2196/60141 (PMC12140504; doi:10.2196/60141)
Supplement: Multimedia Appendix 6 [file diabetes-v10-e60141-s006.docx]

Appendix 6. Statistical performance of different risk prediction approaches.

|  | **Threshold^a^** | **Sensitivity** | **Specificity** | **Positive Predictive Value** | | **NNE^b^** | **% Flagged^c^** |
| --- | --- | --- | --- | --- | --- | --- | --- |
| Simplified Super Learner | 30% | 0.08 | 0.997 | 0.725 | | 1.4 | 0.9 |
|  | 40% | 0.052 | 0.999 | 0.774 | | 1.3 | 0.5 |
|  | 50% | 0.03 | 0.999 | 0.737 | | 1.4 | 0.3 |
| Naive Logistic Regression | 30% | 0.327 | 0.963 | 0.31 | | 3.2 | 5.1 |
|  | 40% | 0.256 | 0.977 | 0.362 | | 2.8 | 3.4 |
|  | 50% | 0.206 | 0.985 | 0.407 | | 2.5 | 2.4 |
| 1. a. The threshold is the predicted risk for an adverse event at which an alert would be issued. | | | | |  |  |  |
| 1. Number Needed to Evaluate (1/PPV) | | | | |  |  |  |
| 1. Percent of individuals with an event who would be flagged at the given threshold | | | | |  |  |  |
